# Study on the Effect of Geometrical Parameters of a Hexagonal Trigger on Energy Absorber Performance Using ANN

**DOI:** 10.3390/ma14205981

**Published:** 2021-10-11

**Authors:** Michał Rogala, Jakub Gajewski, Marcin Górecki

**Affiliations:** 1 Department of Machine Design and Mechatronics, Faculty of Mechanical Engineering, Lublin University of Technology, 20-618 Lublin, Poland; j.gajewski@pollub.pl; 2 Faculty of Civil Engineering and Architecture, Lublin University of Technology, 20-618 Lublin, Poland; m.gorecki@pollub.pl

**Keywords:** thin-walled structures, crashworthiness, hexagonal trigger, buckling

## Abstract

Thin-walled structures are commonly used as energy absorbers in motor vehicles. Their function is to protect the structural components of vehicles and to absorb energy completely during collisions up to 20 km/h. This paper focuses on maintaining crush axiality during research. To verify the numerical analyses, physical specimens were made and then subjected to dynamic crushing. Force and shortening values as well as high-speed camera images were used for data analysis. Through time-lapse shots, plastic deformation within the crush initiator was observed. Such detailed analysis allowed the determination of the influence of hexagonal triggers in the form of notches on the post-buckling progressive analysis. In this paper, neural networks were used to examine the importance of each variable. Data from numerical analyses were used for this purpose. Based on the analyses performed, the effects of both the width and height of the triggers on the crush load efficiency (CLE) and total efficiency (TE) ratios can be seen. The width of the crush initiator has the greatest influence on Crash-box performance. Nevertheless, increasing both the height and the width of the initiator can result in crush non-axiality and underperformance of the energy absorber.

## 1. Introduction

Over the years, the phenomenon of energy absorption has become an increasingly popular topic. Already in the early 1960s, theoretical works considering the dynamic crushing of profiles with square, round, and omega cross-sections began to appear [1]. As a result of significant developments in the automotive industry, the use of metal elements has become more common. At the time, it was believed that the more solid the vehicle was, the greater the safety of passengers and transported objects. As a result of Alexander’s pilot studies, a relationship between the shape of the energy-absorbing element and its performance and overloading during a crash was recognized. Initially, the first Ford vehicles used a steel bumper attached to the stringers—the structural members of the vehicle. These created an apparent sense of safety; however, their energy-absorbing performance was negligible. Over the years, the technology and the state of the art in passive safety increased, and thin-walled systems began to be used in motor vehicles, which were designed to fail in a well-defined manner and absorb as much energy as possible. In the 1980s, research on energy absorption began to appear in the international literature [2]. Many works on the analysis of the behavior of thin-walled sections with round and square cross-sections were developed by Wierzbicki, Chen, Langseth, and Abramowicz [3,4,5]. The elements protecting the stringers—known asenergy absorbers—take on different cross-sections, and this is closely related to the nature of the crushing process. For example, thin-walled profiles with a square section show more stable crushing than those with a circular section, but they achieve a higher peak crushing force (PCF) value, which reduces crushing efficiency [6,7]. This is due to the plastic hinges, which dissipate energy during crushing. In addition, in the case of tubular components, asymmetrical crushes, called diamonds, can occur in some crushing stages—for example at low velocities [8]. Such profiles are also more prone to global buckling during lateral crushing [9].

The description presented by Jones [10] contributed to the development of passive safety as well as to the analysis of thin-walled structures for their performance. With the introduction of the scheme of the destruction of thin-walled profiles, scientists began to influence the shape of the structures studied in order to force their specific behavior [11,12]. They started to use corrugations on the sidewalls, called triggers, to initiate the crushing process in a specific location. The location of the crush initiator also affected the number of plastic hinges produced and thus the efficiency of the total profile. Over the years, scientists have decided to strengthen the structure by using fillings inside the profile to increase the efficiency of energy absorbers. At the turn of the century, Hannsen [13,14] worked on the use of porous structures for absorbing additional energy. Aluminum foam with a certain density and internal structure was used in the research [15,16]. Porous materials exhibit good energy-absorbing properties at low specific weights due to their properties, such as open or closed pores and permeation with other materials and structures [17,18]. Depending on doping with other elements during the production process, we can easily control their stiffness, and thus, their energy absorption capacity.

Thin-walled structures can take a variety of cross-sections—i.e., square, round, or omega [19,20,21,22,23]. Many of these show a positive effect during dynamic crushing; however, they are more expensive to manufacture, and at the point of joining of individual walls, the material often cracks, which decreases the absorber efficiency. Circular and rectangular shapes prove to be the most effective; hence, research has begun to develop profiles of this type with varying sidewall thicknesses [24,25]. The use of thickness gradations allows the stiffness to vary at different stages of the crush, and the energy absorber performance increases as the analysis proceeds [26].

Another way to reinforce the thin-walled structure is through multi-cell filling [27]. The construction and principle of operation of this technique are based on increasing the stiffness in the direction of impact—but by using thin walls inside the profile, scientists have shown that the total efficiency of the absorber is increased, with just a little more maximum force detected during the crushing process [12]. A similar, frequently used solution is honeycomb filling [28,29]. The principle is almost identical to that of multi-cell models; the filling increases the stiffness in the direction of impact so that, although the mean crushing force (MCF) is increased, the PCF also increases significantly, which has a negative effect on the possible overload during the crush. Such solutions, due to their manufacturing difficulty, are rarely found in the automotive or aviation industry. For more geometrically complicated models, neural networks are often used [30,31,32,33], especially regression analysis, which aims to determine the relationship between input and output variables. Structures containing thin-walled elements may be damaged by other types of loading, e.g., cyclic loading—the consequences of which may be disastrous for the load-bearing capacity of the structure. The effects on load-bearing capacity and energy absorption in these cases are described in the following papers [34,35,36,37]. Nowadays, the finite element method is widely used in analysis; with the help of its advanced solvers, it is possible to calculate complex models using fracture of materials or their deformation during dynamic loading modes [38,39,40,41,42].

For the crush analysis of thin-walled profiles, force-displacement diagrams are most commonly used. The most basic quantity determined is the amount of energy absorbed during the crush. It is a measure of the ability of an energy absorber and is also used for further analysis using this quantity. It is referred to as an integral of the characteristic function in the shortening range on which the crushing takes place, and can be determined by the following Equation (1):

(1) $$\mathrm{EA}\left(\mathrm{dx}\right)=\int_{0}^{\mathrm{dx}}F\left(x\right)\mathrm{dx}$$

The mean crushing force is determined using the absorbed energy and the distance over which the crushing occurred. It is determined by the following equation:

(2) $$\mathrm{MCF}=\frac{\mathrm{EA}\left(\mathrm{dx}\right)}{\mathrm{dx}}$$

**Figure 1:**
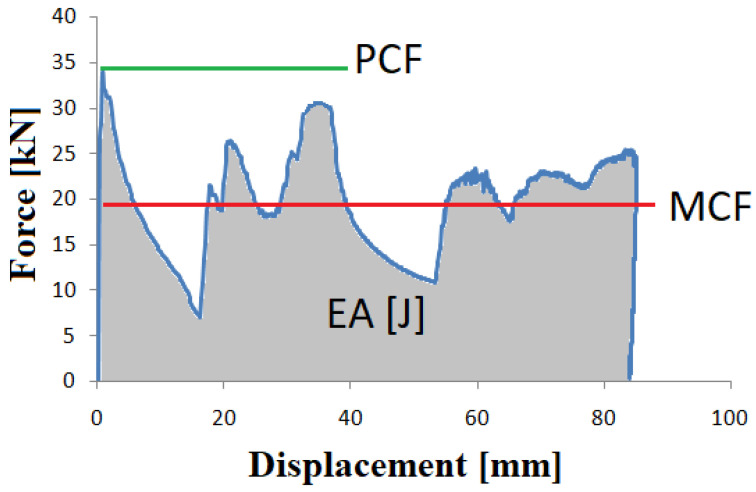
The force-shortening curve for the square energy absorber.

The characteristics presented in these equations show the course of the dynamic analysis; moreover, they allow us to approximate the value of the MCF and the PCF (Figure 1). Knowing these two basic values allows us to estimate the crushing load efficiency (CLE) by dividing the above-mentioned forces. This quantity is the basic parameter determining the crush and can be represented by the following Equation (4).

(3) $$\mathrm{CLE}=\frac{\mathrm{MCF}}{\mathrm{PCF}}*100\%$$

The stroke efficiency (SE) indicator shows the shortening of the specimen that has occurred with reference to the initial length value. Additionally, it is necessary to determine the total efficiency as shown in Equation (5).

(4) $$\mathrm{SE}=\frac{U}{L_{o}}$$

The total efficiency (TE) index is a dimensionless quantity that results from the product of CLE and SE, the indices responsible for the efficiency of the destructive force and specimen shortening, respectively. Using a single indicator, we are able to estimate the crashworthiness of the specimen:

(5) TE=CLE×SE

In the presented studies, neural networks were used to analyze the crushing efficiency indicators and determine which of the geometric parameters have a greater impact on the obtained data. Based on the above indices (Equations (1)–(5)), a multilayer perceptron (MLP) network was modeled, which, by means of specifying input data and output data after specifying appropriate weights, describes the relationships between the various quantities [43].

## 2. Analytical Equations

**Figure 2:**
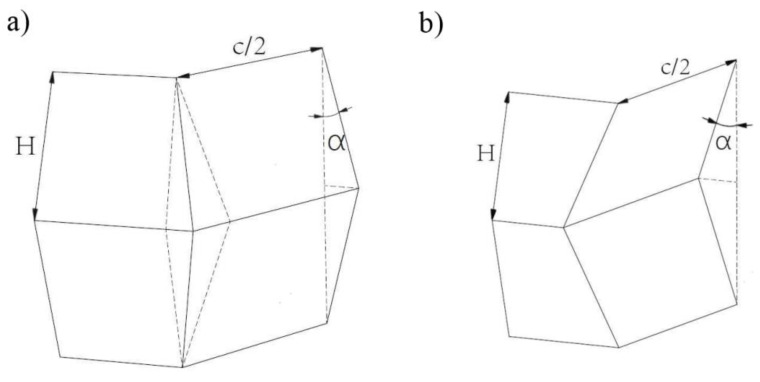
Dynamic progressive buckling of column: (**a**) asymmetrical; (**b**) symmetrical.

The basic quantities for analyzing the energy absorption properties can be determined using geometric relationships. The method of destruction is shown in the following graphics. During local buckling, an important aspect is of the form in which buckling occurs—i.e., in a symmetric or asymmetric way. Both forms are shown below in Figure 2:

Due to the fact that only symmetrical forms of buckling occur during numerical and experimental analyses, all analytical equations presented in this paper apply to such behavior of thin-walled sections. The first quantity that can be derived is the energy absorbed by the folding element, described in Equation (6):

(6) $$E=M_{0}\left(16HI_{1}\frac{b_{f}}{t}+2\pi c+4I_{3}\frac{H^{2}}{b_{f}}\right)$$

By analyzing the effectiveness of the crush distance, it can be assumed that in symmetrical collapse mode:

(7) $$\frac{\delta_{c}}{2H}=0.77$$

In order to determine the value of the MCF, it is necessary to determine the relationship describing the plastic deformation coefficient. For this purpose, the uniaxial Cowper–Symonds constitutive equation was used:

(8) $$\frac{\sigma_{d}}{\sigma_{s}}=1+{\left(\frac{\dot{\varepsilon }}{C}\right)}^{\frac{1}{P}}$$

The strain rate in axial crushing of thin-walled square sections was estimated as:

(9) $$\dot{\varepsilon }=\frac{tv_{m}}{2b_{f}\delta }$$

where *δ* is the crush distance and $v_{m}$ is the average velocity of the tup, which is defined as the initial velocity divided by two.

The magnitude $b_{f}$ is the radius of curvature, which is derived from the shape of the surface and can be represented by the following Equation (10):

(10) $$\frac{b_{f}}{h}=0.53{\left(\frac{c}{t}\right)}^{\frac{1}{P}}$$

Considering the equations mentioned above, the theoretical mean crushing force during dynamic deformation can be determined as follows:

(11) $$\frac{F_{m}^{d}}{M_{0}}=52.22(1+(0.33{\left(\frac{V}{cC}\right)}^{\frac{1}{P}}){\left(\frac{c}{t}\right)}^{\frac{1}{3}}$$

where: $M_{0}=\sigma_{0}\frac{t^{2}}{4}$.

During crash analysis, a very important aspect is the form of the buckling, which affects the profiles’ ability to absorb a certain amount of energy. The length of the semi-wave is closely related to the width of the profile and the thickness of the sidewall, and is described by Equation (12) below [44]:

(12) $$\frac{H}{t}=0.99{\left(\frac{c}{t}\right)}^{\frac{2}{3}}$$

## 3. Numerical Study

**Figure 3:**
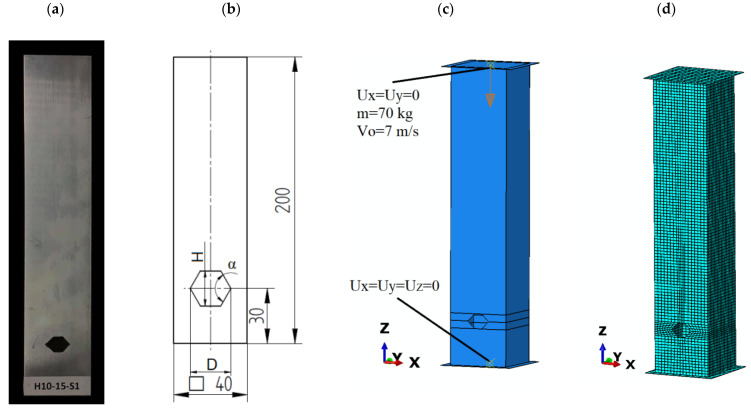
Frontal view of the thin-walled profile: (**a**) experimental specimen (unit: mm); (**b**) dimensioned technical draw; (**c**) boundary condition; (**d**) numerical discretized model.

Numerical simulations were performed using the finite element method in Abaqus software (Abaqus 2019, Dassault Systemes Simulia Corporation, Velizy Villacoublay, France). The study involved aluminum specimens (Table 1) of a square cross-section with a trigger cut on the two transverse walls of the column. The boundary conditions were modeled using non-deformable “Discrete Rigid” plates to allow readout of force as well as displacement values (Figure 3). The aluminum profile was attached to the plate using a tie contact relationship. This relationship does not block the rotational degree of freedom of the profile, so it does not artificially stiffen the structure, whereas the conditions are very close to those of the empirical tests. 

The shape of the initiator was a hexagon, whose geometric parameters are widths from 15 mm to 35 mm in 5 mm increments, and heights from 10 mm to 25 mm in 5 mm steps (Table 2). 

The side edges of the cutout formed an α angle, as shown in Figure 3b. The distance of the initiator from the bottom edge was 30mm and was the same for all the samples tested.

The numerical analysis was conducted in two stages. The first stage was to load the profile with unit axial force to obtain the buckling form, which corresponds to the first mode of frequency. Then, the profile geometry with the implemented buckling form was dynamically loaded with a mechanical energy of 1700 J. The energy was defined by assigning a mass of 70 kg to the top plate serving as a tup and giving it an initial velocity of 7 m/s (Figure 3c). The mesh size is shown in Figure 3d; for the profile it was two, and for the bottom plate and top plate it was five due to their negligible impact during the analysis. The values were chosen so that the results obtained were as accurate as possible with reasonable computational time. All analyses were carried out until the tup insert lost its total velocity and the force values read at the base of the profile reached zero. Based on the obtained force and shortening courses with the use of Equations (1)–(5), the crushing efficiency ratios were calculated.

**Figure 4:**
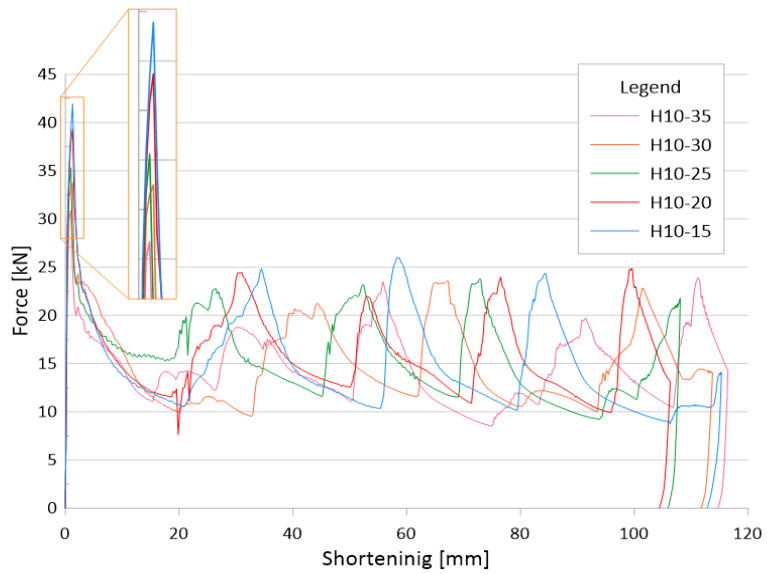
Force waveforms for all samples with a trigger height of 10 mm.

**Figure 5:**
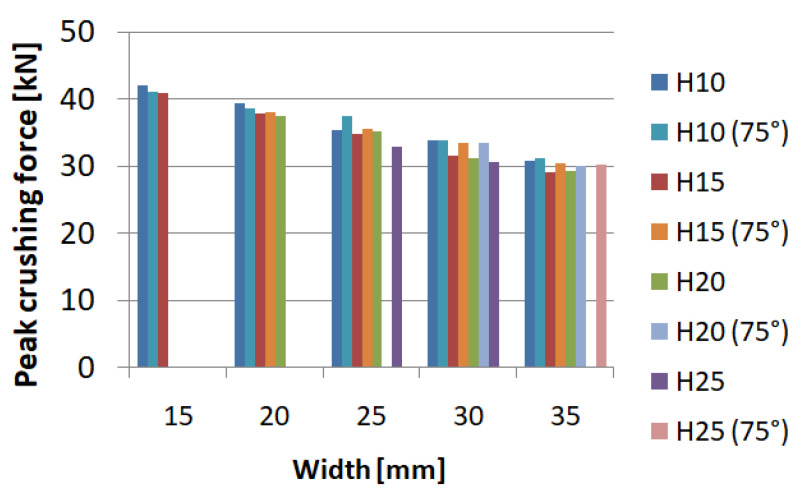
Value of peak crushing force for the model with hexagonal trigger.

The force-shortening graph (Figure 4) shows the change in characteristic curves for different widths of the crush initiator at a fixed height of 10 mm. The graph does not show results for all initiator heights so as not to cloud the diagram. The above characteristics also show a large difference in the total shortening of the specimens that occurred during the crush. The force peaks that occurred at the beginning of the crush are detailed in the graph, the change in force can also be observed in Figure 5. 

Figure 5 shows the values of the maximum force obtained during the crush. For all samples, the force was generated at the beginning of the crush and is directly related to the shape of the trigger and its geometric parameters. Observing the data, it can be noticed that the value of PCF decreases with the increasing width of the notch, as well as with the height of the initiator. For extreme cases, we saw a decrease in force value by as much as 25 percent, while for specimens of the same width, a decrease of up to about 10 percent was noticeable, which significantly reduced the overload occurring during the crush. 

**Figure 6:**
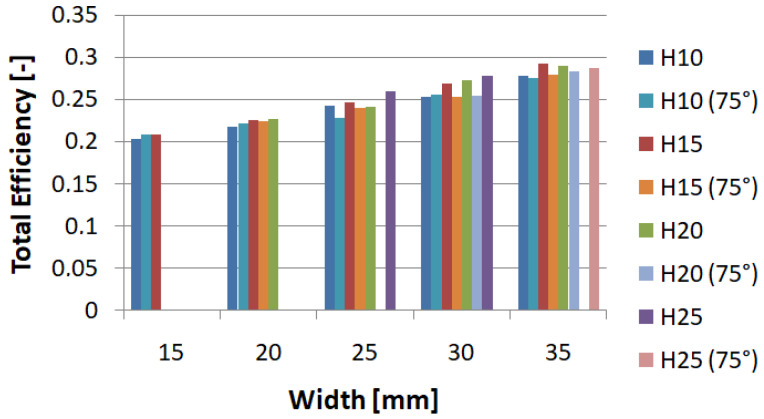
Value of the total efficiency indicator for the model with hexagonal trigger.

Some of the values were not determined due to geometric constraints, since the trigger cannot be higher than it is wide; therefore, only two values were determined for the width of 15 mm. The highest ratio was found for the models with the largest width, 35 mm, over the whole range of trigger heights (Figure 6). However, for individual width groups, an increasing tendency could be seen as the initiator height increased. For the models examined, an increase of up to 50% could be seen.

**Figure 7:**
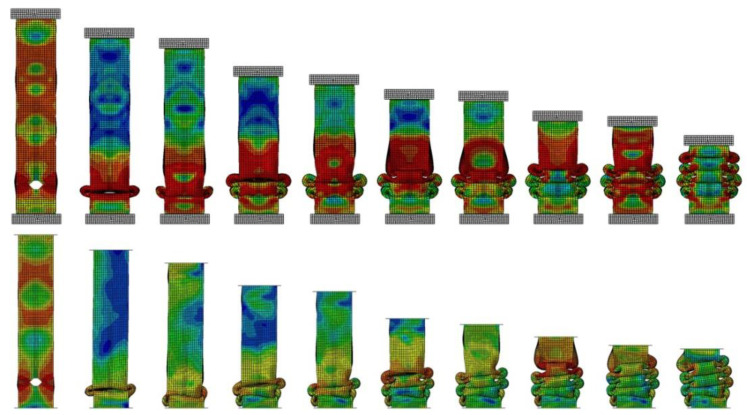
Comparison of mode for numerical simulation of the H10-15 specimen with and without skewness.

Figure 7 shows the failure stages of thin-walled sections where local buckling and subsequent fold compression occurred. Two numerical analyses are shown in the figure, where one shows axisymmetric behavior and the other shows a slight skewing of the specimen. This occurs due to the behavior of the first fold, where the material on the right side of the column curls upward and the material on the left side drops downward. Similar behavior in the specimen was observed during the experimental study. Both samples had identical mesh densities. The crush efficiency ratios determined from the characteristics of the two analyses are shown in Table 3.

By comparing the indicators of two specimens with identical geometry and initial conditions, a slight discrepancy in the values obtained could be observed. The difference was seen in the maximum initial force, as well as the MCF values. The specimens showed slightly different behavior and fold formation, but the CLE values remained almost identical.

## 4. Experimental Study

Experimental tests were carried out on a sample of specimen H10-15. The results were used to validate the data obtained numerically. The aluminum profile with a hexagonal cut-out is a machine-manufactured, so the results of the shape of the cutout have a certain level of accuracy. Two copies of the test specimen were made in order to confirm the force waveform during dynamic post-buckling analysis. The experimental analysis was performed on an INSTRON CEAST 9450 HES machine (Norwood, MA, USA).

**Figure 8:**
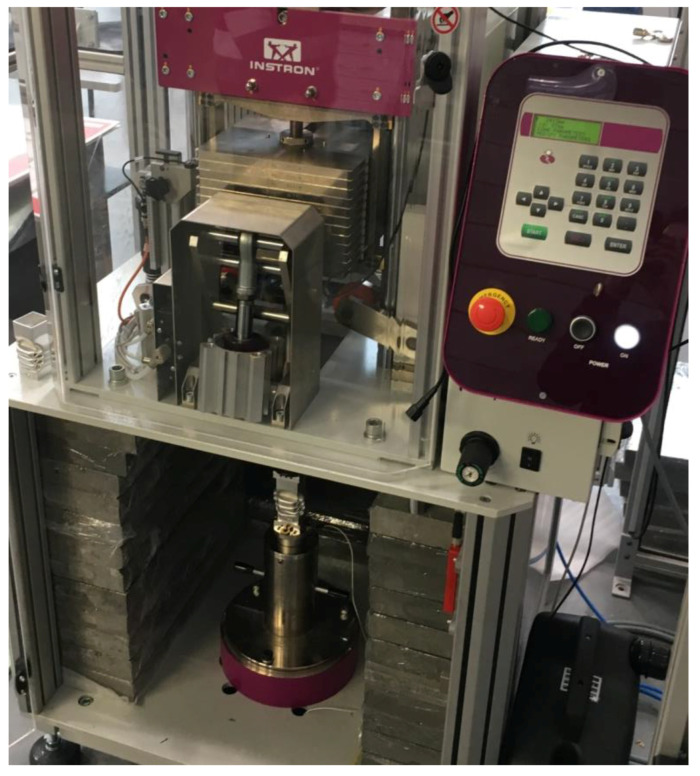
General view of dynamic testing machine Instron Ceast 9450 HES.

Figure 8 shows a dynamic testing station with a crushed specimen attached to the test table. The center of the trigger was 30 mm from the bottom edge. The model fixing was done with push-on aluminum cubes. The entire specimen, including the fixture, was attached to the test table. 

**Figure 9:**
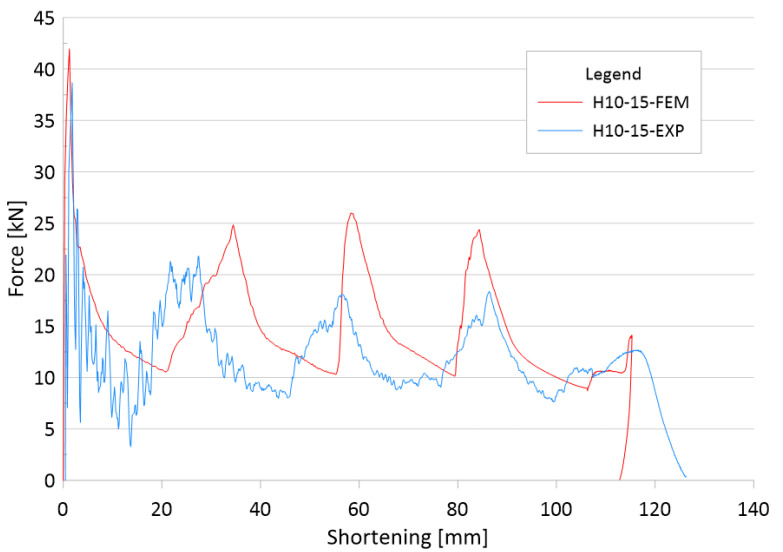
Comparison of numerical and experimental force-shortening characteristics.

**Figure 10:**
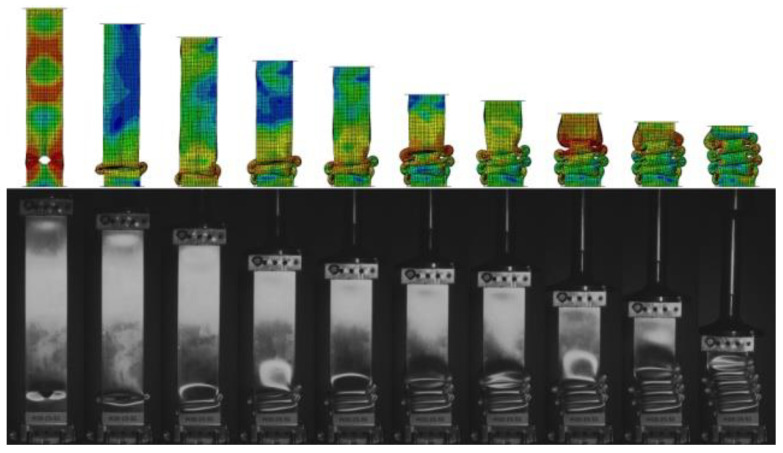
Modes of crushed of H10-15 thin-walled specimen.

Based on Figure 9 and Figure 10, we can observe the behavior of the specimen when subjected to a dynamic axial force. In the graph, we can see the fitting of the experimental and numerical curves, which allows us to determine the location of the plastic hinges and the value of the force generated during its formation. In addition, we can see the total shortening of the specimen and the value of the maximum failure force, which are very helpful in studying dynamic crushing performance.

The comparison presented in Figure 8, correlates with the force-shortening graph. In the experiment, we used a high-speed camera PHANTOM MIRO 310 (Phantom, NJ, USA)—thanks to which we can observe the locations of the fold formations and their size, and compare them with those obtained during the numerical analysis. This is an extremely valuable tool for observing the nature of the course of failure of thin-walled sections.

## 5. Neural Network Analysis

The network consists of multiple neurons arranged in layers. Each neuron calculates a weighted sum of its inputs, and the excitation level thus determined becomes the argument of the transition function (activation function), which calculates the output value of the neuron (Table 4) [45]. Neurons form a unidirectional structure, that is, the transmission of signals takes place in the direction from input to output—without feedback. The number of input and output neurons is determined by the problem being solved [46].

**Figure 11:**
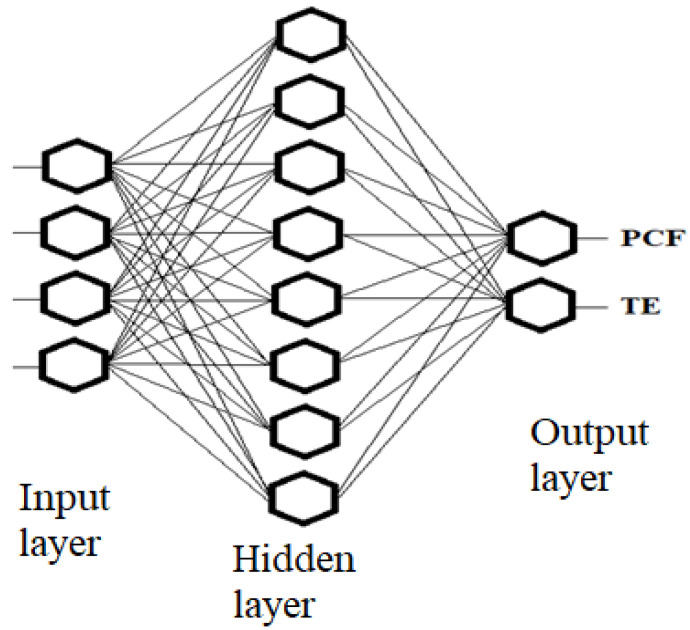
Graphical visualization of the applied neural network.

The above neural network (Figure 11) consisted of two qualitative and quantitative variables. The qualitative variable is the angle, hence, two inputs in the network were assigned for 75° and 90° angles. The quantitative variables were the width and height of the crush initiator [47]. The neural network had eight neurons in the hidden layer and two characteristic crush quantities in the output PCF and TE.

In the analyzed case, the network input values were geometric parameters such as width and height, and they were defined as quantitative variables, while the changing angle, i.e., 75 and 90, was a qualitative variable; hence, there are four input values in the network description. The network had eight neurons in the hidden layer and two variables at its output, i.e., the amount of PCF force and TE indicator.

**Figure 12:**
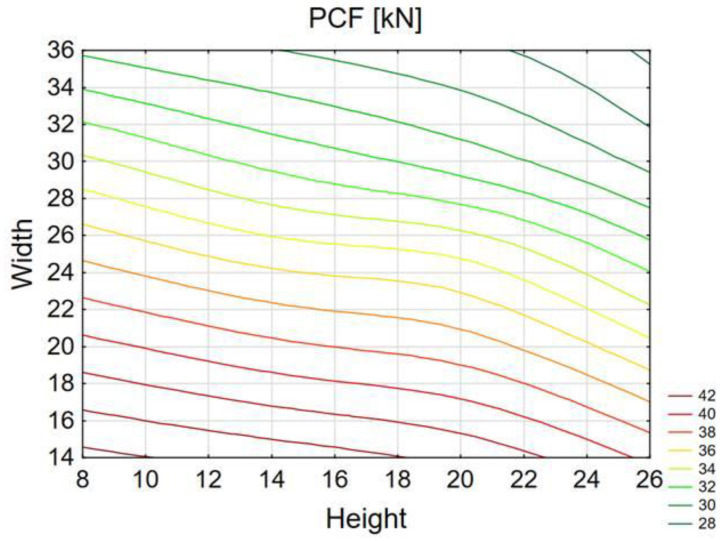
Predicted PCF value for the different widths and heights of the initiator.

The values obtained by using neural networks coincided with those examined by the finite element method. The PCF force in particular changed its value as the initiator width changed (Figure 12). The magnitude of the PCF did not show much variation with changes in trigger height.

**Figure 13:**
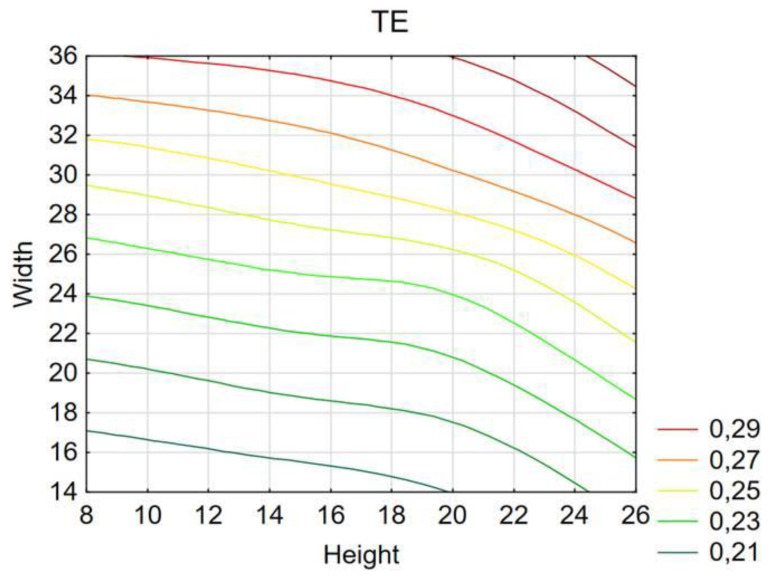
Predicted TE value for the different widths and heights of the initiator.

The above graph (Figure 13) shows the TE index values obtained using the MLP (multi-layer perceptron) network. The network had very high rates of learning, testing, and validation, which proves the reliability of data. The plane diagram shows that the highest performance was achieved by models with large width and high initiator height. The models achieved high performance for models with an initiator width over 30mm over the whole height range.

The last analysis was on sensitivity to particular geometrical parameters. The analysis took into account the height, width, and angle of the arms extended. The analysis, giving the result of more than one parameter, states the usefulness of each particular one. Based on the data presented in Table 5, we can see that the width of the initiator had the biggest influence on the results; the second biggest was the arm-opening angle.

## 6. Conclusions

Dynamic crush testing is a very important aspect of passive safety analysis. The forces generated during the crush are reflected in the amount of overload. Reducing the initial force is especially important during the design process of energy absorbers. Reducing the initial force has an impact on the efficiency of the crushing force and thus indirectly on the total efficiency of a thin-walled structure.

In this study, dynamic crushing of square section columns with hexagonal cut-out as an initiator was considered. Based on the obtained numerical data, crush indices were determined. Special attention was paid to the improvement of energy absorption indicators, such as the CLE or TE of the specimen, during dynamic testing. In numerical analyses, most of the works show complete symmetry of the dynamic crush behavior, which is usually impossible to obtain during empirical tests; thus, attention was also paid to the sample skewness that occurs during post-stroke analysis. This paper presents a comparison of numerical analyses with a dynamically tested specimen; the determined indices were compared with each other. In addition, the ratios determined from the experimental analysis as well as the time-lapse film analysis allow us to conclude that the results using the finite element method are reliable. The study showed a positive effect of using a hexagonal trigger on the behavior of the thin-walled structure. A decrease in PCF of up to 25% was observed for models with a trigger width of 35 mm. The total efficiency increased linearly and reached a maximum increase of up to 50%. Moreover, the studied groups of models were analyzed for two trigger edge inclination angles. The study showed that the width of the crush initiator has the greatest influence on structure behavior. To a much lesser extent, changes in energy-absorption indices are affected by changes in height (H) and inclination angle (α).

Therefore, the models allow for greater structural safety with the same geometric dimensions of the specimen by changing only the dimensions of the crush initiator. 

Dozens of models were examined to determine the effect of individual dimensions on the total efficiency. In addition, the study was enriched with analysis using neural networks. The sensitivity analysis also showed that the second most important factor is the angle of inclination of the side edges, so it is worth taking this into consideration while designing triggers. Models with higher initiator heights showed good overall performance, but due to the size of the notch the profiles may show instability during dynamic crushing.

## Figures and Tables

**Table 1:** Materials properties of the aluminum columns Data from Ref. [26].

AA-6061 Aluminum Material Properties	
Density [kg/m^3^]	2700
Young Modulus [GPa]	70
Poisson ratio v [-]	0.33
Yield Strength R_e_ [MPa]	200
Tensile Strength R_m_ [MPa]	279.98
Elongation A% [%]	5.98

**Table 2:** Range of geometric dimensions of the hexagonal cut-out trigger.

	Scope	Spacing [mm]
Height (H)	10–25 [mm]	5
Width (D)	15–35 [mm]	5
Angle (α)	75, 90 [°]	-

**Table 3:** Crashworthiness indicator of two different crush types in the example of the H10-15 model.

Crush	EA [J]	U2 [mm]	PCF [kN]	MCF [kN]	CLE [-]	STE [-]	TE [-]
Skew	1705.3	116.8	43.239	13.453	0.311	0.634	0.197
Axial	1712.6	122.4	40.848	12.935	0.317	0.662	0.210
Difference	0.43%	4.26%	5.85%	4.00%	1.75%	4.26%	5.93%

**Table 4:** Quality of the MLP neural network for the absorber with a hexagonal trigger.

	PCF Learning	PCF Testing	PCF Validation	TE Learning	TE Testing	TE Validation
MLP 4-8-2	0.981934	0.999449	0.992027	0.983720	0.998301	0.976134

**Table 5:** Sensitive analysis of geometrical parameters of crush initiators.

Network	Width	Angle	Height
MLP 4-8-2	23.10155	2.105311	1.439345

## Notation

*u*	*shortening of specimen*
*L_o_*	*initial height of a specimen*
$\sigma_{d}$	dynamic yield stress
$\sigma_{s}$	static yield stress
$\dot{\varepsilon }$	strain rate
C,P	constant Cowper–Symonds relation
*H*	*the half distance between adjacent plastic hinges*
*t*	*sidewall thickness*
*c*	*two widths of the profile*
$\delta_{c}\;$	crushing distance
$b_{f}\;$	*radius of toroidal plastic hinge*
*I_1_, I_3_*	*integrals in Equation (5)*
$F_{m}^{d}$	average crushing force for dynamic analysis
$v_{m}$	average velocity of tup
